# Transcriptomic profiling of the yeast *Komagataella phaffii* in response to environmental alkalinization

**DOI:** 10.1186/s12934-023-02074-6

**Published:** 2023-04-04

**Authors:** Marcel Albacar, Abdelghani Zekhnini, Jorge Pérez-Valle, José L. Martínez, Antonio Casamayor, Joaquín Ariño

**Affiliations:** 1 Institut de Biotecnologia i Biomedicina & Departament de Bioquímica i Biologia Molecular, Universitat Autònoma de Barcelona, Cerdanyola del Vallès, 08193 Spain; 2 Department of Biotechnology and Biomedicine, Section for Synthetic Biology, Technical University of Denmark, Kongens Lyngby, Denmark

**Keywords:** Stress response, Oxidative stress, Promoter regulation, Protein production

## Abstract

**Background:**

Adaptation to alkalinization of the medium in fungi involves an extensive remodeling of gene expression. *Komagataella phaffii* is an ascomycetous yeast that has become an organism widely used for heterologous protein expression. We explore here the transcriptional impact of moderate alkalinization in this yeast, in search of suitable novel promoters able to drive transcription in response to the pH signal.

**Results:**

In spite of a minor effect on growth, shifting the cultures from pH 5.5 to 8.0 or 8.2 provokes significant changes in the mRNA levels of over 700 genes. Functional categories such as arginine and methionine biosynthesis, non-reductive iron uptake and phosphate metabolism are enriched in induced genes, whereas many genes encoding iron-sulfur proteins or members of the respirasome were repressed. We also show that alkalinization is accompanied by oxidative stress and we propose this circumstance as a common trigger of a subset of the observed changes. *PHO89*, encoding a Na^+^/Pi cotransporter, appears among the most potently induced genes by high pH. We demonstrate that this response is mainly based on two calcineurin-dependent response elements located in its promoter, thus indicating that alkalinization triggers a calcium-mediated signal in *K. phaffii*.

**Conclusions:**

This work defines in *K. phaffii* a subset of genes and diverse cellular pathways that are altered in response to moderate alkalinization of the medium, thus setting the basis for developing novel pH-controlled systems for heterologous protein expression in this fungus.

**Supplementary Information:**

The online version contains supplementary material available at 10.1186/s12934-023-02074-6.

## Background

Adaptation to changes in the pH of the environment is critical for survival of yeast cells. The mechanisms underlying such adaptation are largely mediated by remodeling of the transcriptional response and have been investigated with substantial detail in fungi such as *Saccharomyces cerevisiae*, *Candida albicans*, and *Aspergillus nidulans* [1–4]. Because adaptation to niches with diverse pHs appears critical for fungal pathogenesis, pH signaling mechanisms have been proposed as possible antifungal targets [5]. Major signaling pathways responsible for this response include the conserved PacC/Rim101 pathway, the calcineurin phosphatase pathway, mediated by the transcription factors Crz1/CrzA, or the protein kinase A (PKA) pathway.

The PacC/Rim101 pathway is remarkably conserved among fungi and is based in a signaling pathway (named the Pal pathway in filamentous fungi, and the Rim pathway in yeast-like fungi) that is triggered by the activation of a 7-transmembrane domain receptor that, ultimately, leads to the proteolytic processing and activation of the transcription factor PacC (Rim101 in *S. cerevisiae*). In *S. cerevisiae*, most of the effects of Rim101 activation are due to the repression of the expression of Nrg1, which in turn acts as a transcriptional repressor [6]. The calcineurin pathway is activated by an increase in intracellular calcium triggered by extracellular alkalinization [7, 8] and results in activation of the protein phosphatase calcineurin. Then, this enzyme dephosphorylates the transcription factor Crz1 and promotes its transit to the nucleus, where it activates a substantial number of genes [7, 9]. The relevance of the PKA pathway in the adaptation to high pH has been also demonstrated, although its role seems to be species dependent. Thus, in *S. cerevisiae*, activation of PKA associates with decreased alkaline tolerance [10], whereas in the human pathogen *Cryptococcus neoformans* is involved in increasing tolerance to high pH [11]. Additional pathways have been identified in *S. cerevisiae* to be activated upon alkalinization, such as the *PHO* regulon that responds to phosphate scarcity [12], the cell wall integrity Slt2/Rml1 pathway [13] or the Snf1 pathway [14]. It is worth noting that in *S. cerevisiae* there are examples (such as *ENA1* or *PHO89*) of promoters containing diverse structural elements that allow the integration of signals mediated by various independent pathways to allow coordinated and timely transcription [15, 16].

*Komagataella phaffii* (formerly called *Pichia pastoris*) is an ascomycetous yeast characterized by an obligatory aerobic metabolism and the ability to use methanol as sole carbon and energy source. This yeast has become an organism extensively used for heterologous protein expression. This popularity derives from a number of advantages, such as the ability to grow at high cellular densities, efficient mechanisms for secretion of produced proteins to the medium, low level of secreted endogenous proteins (which facilitates purification of heterologous secreted products), and a more suitable pattern of glycosylation of secreted proteins [17, 18]. Heterologous protein expression in this organism is very often driven from the powerful *AOX1* promoter, required for methanol utilization [19]. This is a strongly controlled and very potent promoter, but the toxic and flammable nature of methanol causes safety concerns and is considered a limitation in the process. This has led to the search of novel promoters and induction systems that could be considered an alternative [20, 21]. More recently, *K. phaffii* has been proposed as an emerging model organism in biological research, particularly suitable for the investigation of methanol assimilation, mechanism of protein secretion, peroxisome biogenesis and pexophagy, or the machinery for maintenance of cell wall integrity [22].

Despite the relevance of this organism, the knowledge about the mechanisms activated in response to stress are, in comparison with other yeast such as *S. cerevisiae* or *S. pombe*, relatively scarce. This is a significant limitation, since there are growing evidence that activation of stress responses positively influences the expression of recombinant proteins [23, 24]. In particular, the adaptive mechanisms allowing survival when pH in the environment becomes moderately alkaline have not been examined. In this work, we investigate the changes in the transcriptional landscape of *K. phaffii* occurring in response to sudden alkalinization of the medium. We present evidence that this situation triggers a widespread remodeling of gene expression through mechanisms that likely include the activation of calcineurin signaling. This knowledge constitutes a key step that will guide further research to devise novel promoters able to respond to a very simple and inexpensive stimulus.

## Results

### Alkalinization alters mRNA levels for hundreds of genes in *K. phaffii*

**Fig. 1:**
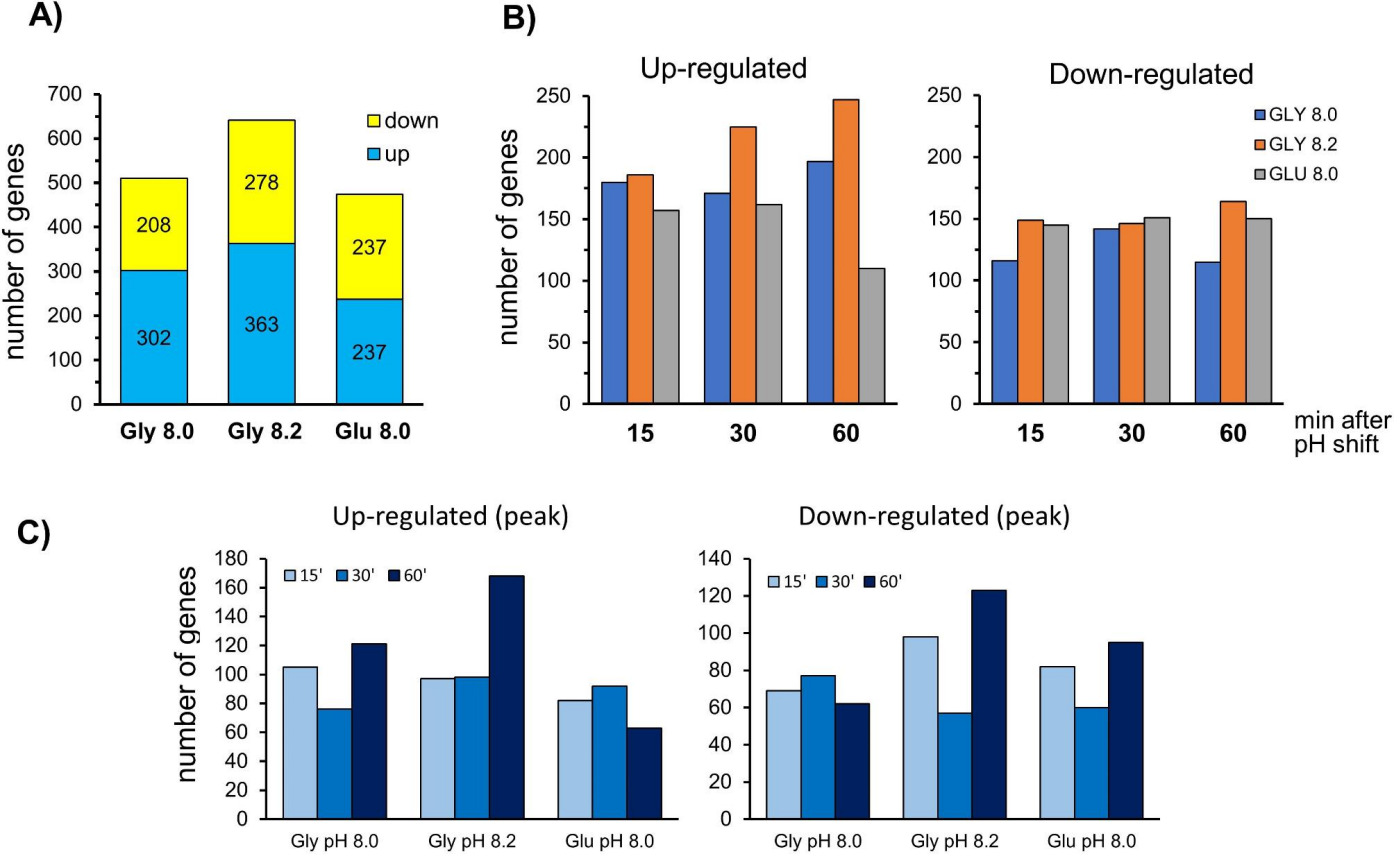
Transcriptional impact of environmental alkalinization in *K. phaffii*. (**A**) Number of genes up-regulated or down-regulated (log2 > = 1 or <= -1 and p-value < = 0.01) for each of the conditions tested. (**B**) Time-course of emergence of up-regulated and down-regulated genes. (**C**) Number of genes showing the stronger expression change (peak) for each time and experimental condition

*K. phaffii* X33 cells exponentially growing on glycerol were shifted from medium at pH ∼5.5 to pH 8.0 or 8.2, whereas similar cultures growing on glucose were shifted to pH 8.0. Samples were taken just before the pH shift and after 15-, 30- and 60-min. RNA-seq analysis showed the expected differential profile, in the absence of stress, between cells grown on glucose compared to those grown on glycerol, namely increased expression of glycolytic genes, and repression of those relevant in gluconeogenesis and glycerol utilization (Additional File 1, Supp. Figure 1). When the impact of alkalinization was examined, we found 772 genes with a change in mRNA levels of at least 2-fold (log_2_ > = 1 or <= -1, with a *p*-value < 0.01) for at least one time or condition (Additional File 2). Of these, 430 were induced and 378 repressed (36 genes were induced under some circumstances and repressed in others). As shown in Fig. 1A, the highest number of changes was observed for cells grown on glycerol and shifted to pH 8.2. The number of affected genes in cells shifted to pH 8.0 was slightly higher for cells grown on glycerol than on glucose, mostly due to an excess of induced genes (302 vs. 237). About 61% of genes induced at pH 8.0 in cells grown on glycerol were also induced in cells grown on glucose, whereas for repressed genes the overlap was of 68%. The transcriptional response was very fast, with over 170 genes up-regulated and nearly 150 genes down-regulated 15 min after shifting cells to alkaline pH (Fig. 1B). In fact, in cells grown on glycerol and shifted to pH 8.0, the value at 15 min was the peak of the time-course for about 35% of both up-regulated and down-regulated genes (Fig. 1C). In contrast, in cells shifted to pH 8.2, the peak was clearly delayed to 60 min, particularly for induced genes (56% of genes).

**Fig. 2:**
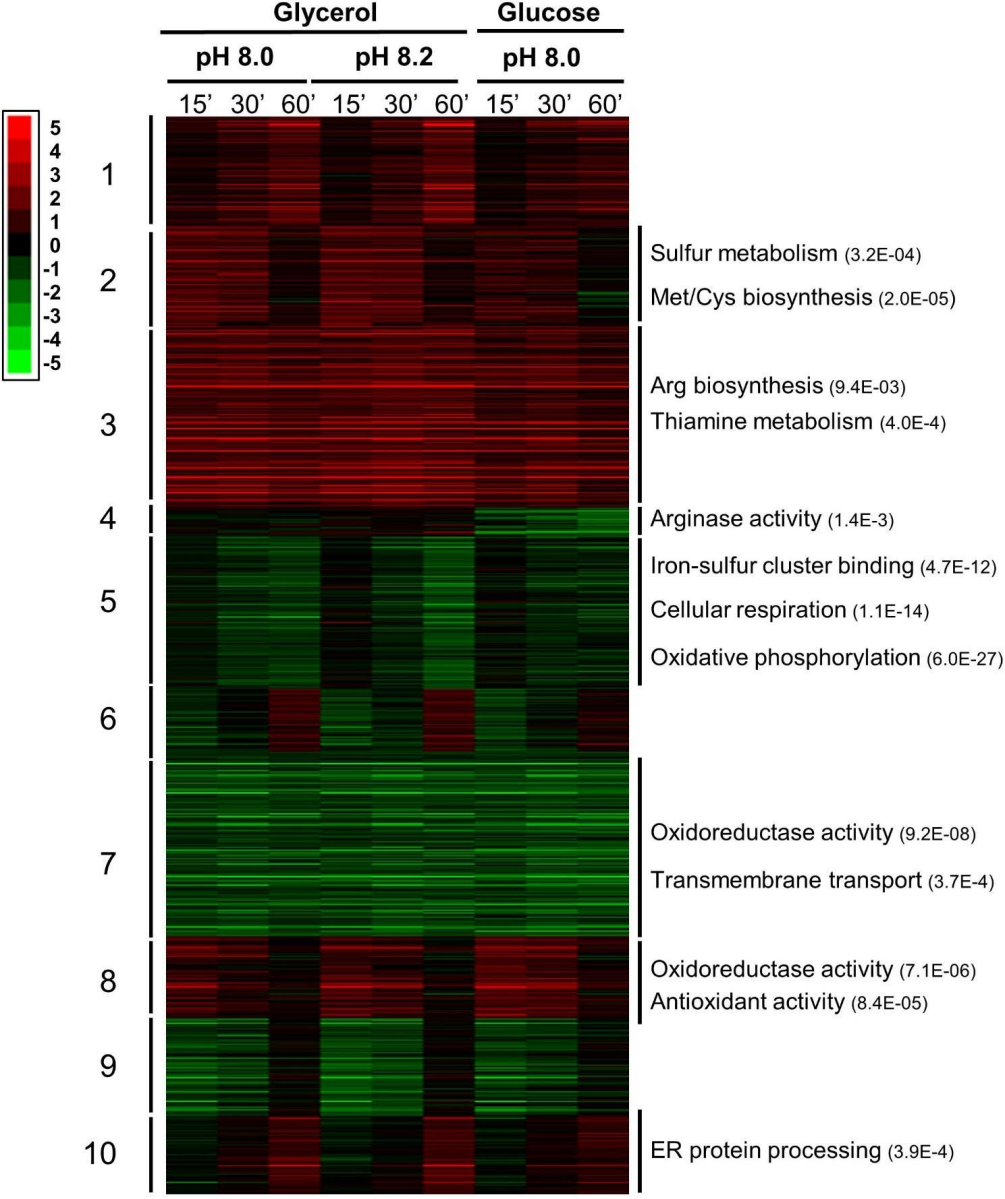
Changes in expression for the 772 induced or repressed genes at least at one time-point and condition. Data was clustered with the Gene Cluster software v.3.0 [60] using k-means algorithm (10 clusters, 100 iterations, uncentered correlation) and visualized using Java TreeView [61]. The most relevant specific GO enrichment terms for the clusters generated are shown with the p-value in parentheses. The intensity of the expression change can be inferred by comparison with the enclosed scale (log2 values)

**Fig. 3:**
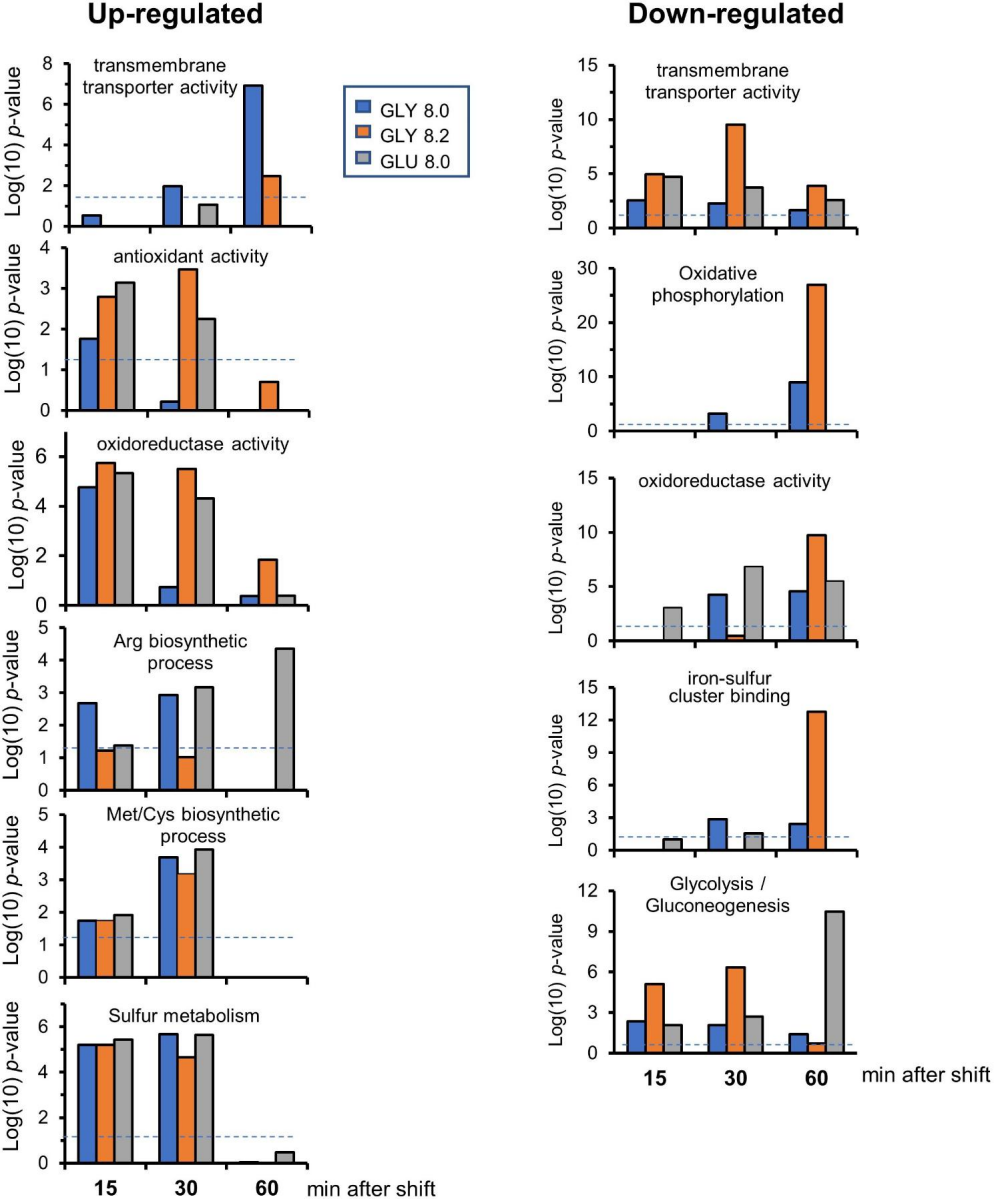
Time course for up-regulated and down-regulated genes for diverse GO terms derived from the entire set of data. The enrichment for each time and condition is expressed as log10 of the *p*-value. The discontinuous line sets the *p*-value < 0.05 threshold

The k-means algorithm was used to define 10 clusters based in the expression profile of the genes (Fig. 2). Gene Ontology analysis of the different clusters revealed several interesting associations. Cluster 2 and cluster 3 were made of induced genes, and the former was enriched in genes involved in sulfur metabolism (mainly Met and Cys biosynthesis), while the latter contained diverse genes related to arginine and thiamine biosynthesis. Conversely, the small cluster number 4, which includes genes particularly repressed in cells grown on glucose, comprises the genes encoding the first and second steps for Arg degradation (two putative Car1 arginases, C4R298 and C4R8L9, and the Car2 (C4R4H3) ornithine aminotransferase). Cluster 5 contains genes generally repressed after 30 and 60 min of stress, and it is highly enriched in genes encoding proteins required for cellular respiration and oxidative phosphorylation, as well as those containing iron-sulfur clusters. Cluster 7 contains genes that code for enzymes with redox activity and involved in transmembrane transport, and they are constantly repressed from min 15 onwards. Genes transiently induced after 15 and 30 min are found in cluster 8, which is enriched in those encoding proteins involved in oxidative stress response and, as in cluster 7, also with redox activity. However, the overall temporal and functional enrichment profile of down-regulated (cluster 7) and up-regulated genes related to redox activity (cluster 8) is very different (Fig. 3), since induction of redox-related up-regulated genes is an early process (typically at 15 and 30 min), whereas repressed genes are enriched after 60 min, particularly for cells shifted to pH 8.2.

### Alkalinization of the medium triggers oxidative stress in *K. phaffii*

**Fig. 4:**
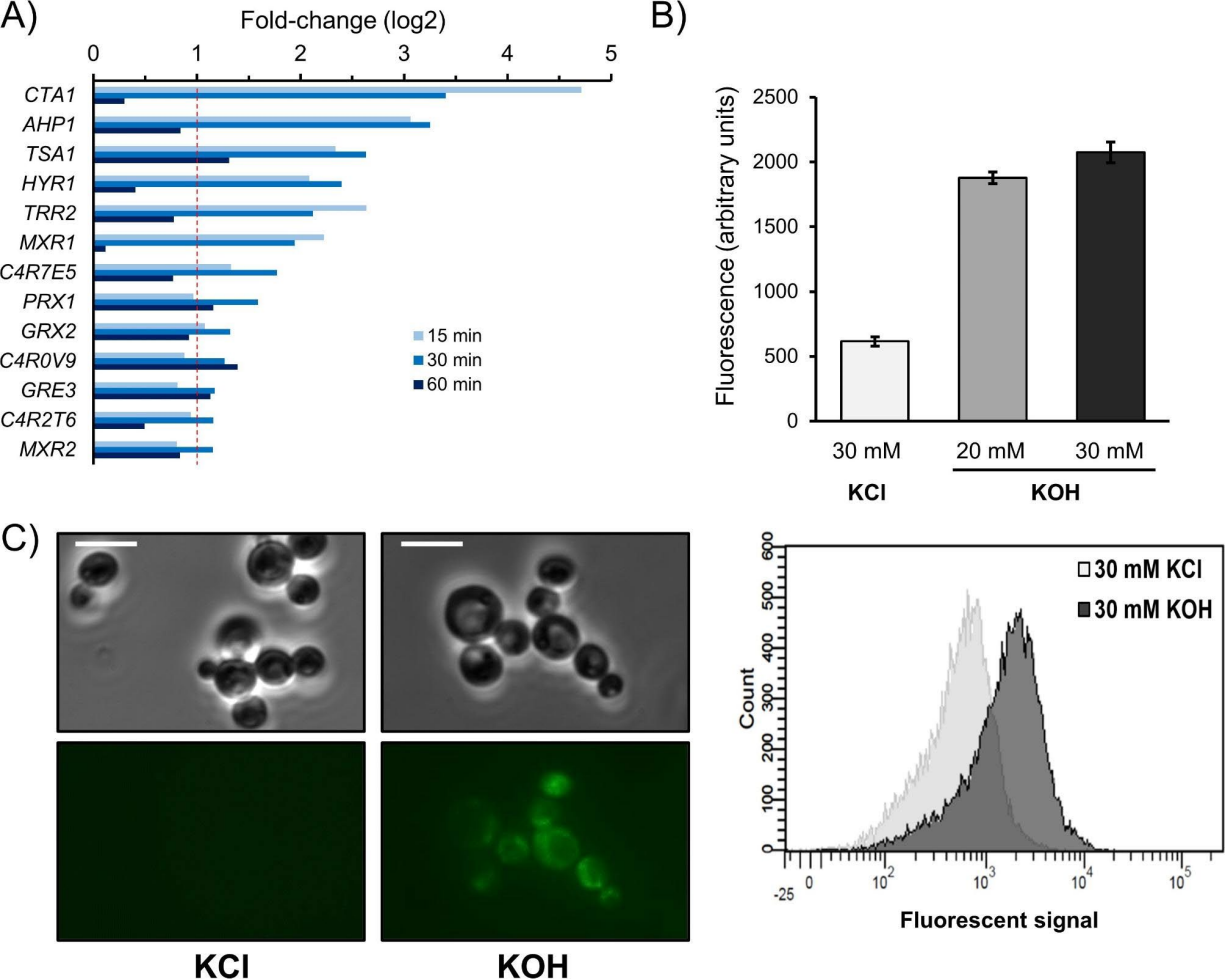
Alkalinization of the medium triggers an oxidative stress response in *K. phaffii*. (**A**) Changes in expression for diverse genes known to be responsive to oxidative stress in yeasts. Data corresponds to cells grown in glycerol and switched to pH 8.2. The discontinuous red line indicates, in this and subsequent figures, the log2 > = 1 and <= -1 thresholds. (**B**) *K. phaffii* X33 cells were grown in YPD medium and incubated for 1 h with dihydrorhodamine 123. Aliquots were taken and treated with KCl (30 mM), or KOH (20 and 30 mM, raising the pH to 7.4 and 8,1, respectively). After 30 min of incubation, cells were processed for flow cytometry. The lower panel shows the fluorescence shift of the population treated with 30 mM KOH. (**C**) Cultures treated with 30 mM KOH) were observed under a Nikon Eclipse TE2000-E using the FIT-C filter. Scale bar denotes 5 μm

The antioxidant response of *K. phaffii* to alkaline pH was further investigated. Figure 4A shows the expression profile of diverse genes induced shortly after pH shifting and encoding proteins widely recognized as involved in the response to oxidative stress. These included C4R2S1 (Cta1, catalase A), C4QXN4 (Ahp1, a peroxiredoxin), C4R2H3 (Tsa1, Thioredoxin peroxidase), or C4R934 (Grx2, a Glutaredoxin). This profile suggested that exposure to alkaline pH triggers a situation of oxidative stress in *K. phaffii*. Such possibility was investigated by incubating cells with the dihydrorhodamine 123 dye, which becomes fluorescent in the presence of reactive oxygen species. As shown in Fig. 4B, exposure of the cells to moderate alkalinization (pH 7.4, by adding KOH up to 20 mM) already triggers a substantial increase in fluorescence that is slightly augmented when pH is raised to 8.1 (30 mM KOH), as documented by flow cytometry analysis. This effect can be also detected by fluorescence microscopy Fig. 4C, revealing that cells become clearly fluorescent after shifting the medium to pH 8.1.

### Impact of alkalinization on the metabolic transcriptome

**Fig. 5:**
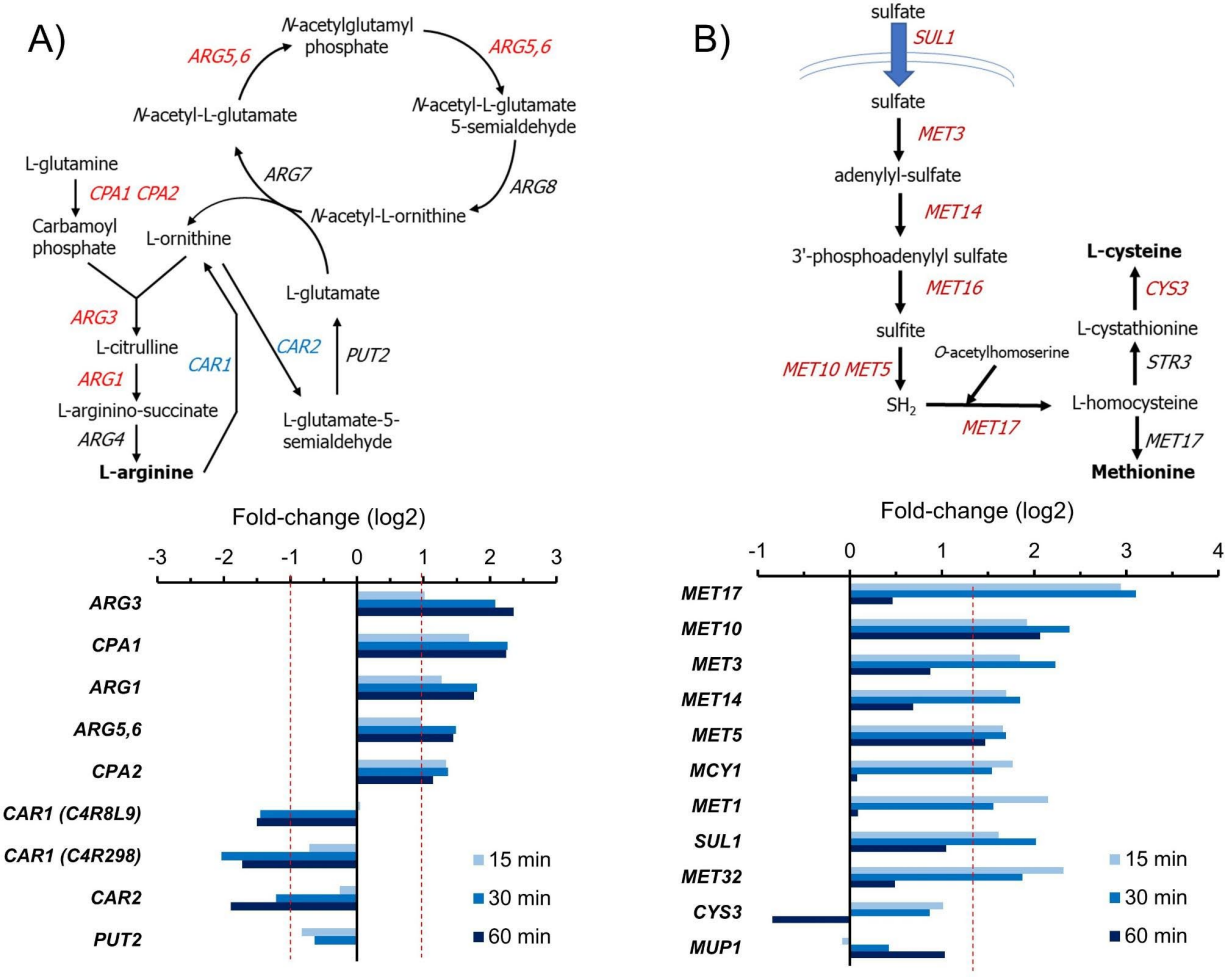
Changes in expression in genes related to the biosynthesis of arginine (**A**) and the metabolism of sulfur and the amino acid cysteine (**B**). Data for A and B correspond to cells grown on glucose or glycerol, respectively, and shifted to pH 8.0. Genes induced are denoted in red and those repressed in blue

As hinted in Fig. 2 (cluster 3) and 3, alkalinization causes the induction of diverse genes encoding proteins required for arginine biosynthesis. These included (Fig. 5A) both subunits of carbamoyl-phosphate synthase (C4R6Y9/Cpa1, and C4R5L7/Cpa2), C4QZV1 (Arg5,6), a bifunctional enzyme that catalyzes two steps in the cyclic transformation of glutamate into N^2^-acetyl-L-ornithine, as well as the consecutive steps catalyzed by C4R533 (Arg3, ornithine carbamoyltransferase) and C4R3W1 (Arg1, argininosuccinate synthase). Interestingly, in cells grown on glucose, the genes related to the steps involved in arginine degradation are repressed (Fig. 2, cluster 4), including two genes encoding putative Car1 arginases (C4R298 and C4R8L9), the one that encodes Car2 (C4R4H3), and even Put2 (C4R1 X 5), albeit the latter does not reach the log2 < = 1 threshold. We also observe a moderate decrease in the expression of genes encoding possible plasma-membrane arginine transporters (Can1/C4QYY6 and Gap1/ C4QYK8).

Genes involved in sulfate assimilation and Met and Cys biosynthesis are also induced upon alkalinization (Fig. 5B), including the ones encoding the sulfate transporter Sul1 (C4R4Q2), the zinc-finger DNA-binding transcription factor Met32 (C4QVF9), and a putative Mcy1 cysteine synthase (C4R122), able to catalyze the conversion of O-acetyl-L-serine into cysteine. As it can be observed, the response was very fast and transient for most genes (note that *CYS3* was even repressed after 45 min). The exception was *MUP1*, encoding a high affinity methionine permease involved in both methionine and cysteine uptake.

**Fig. 6:**
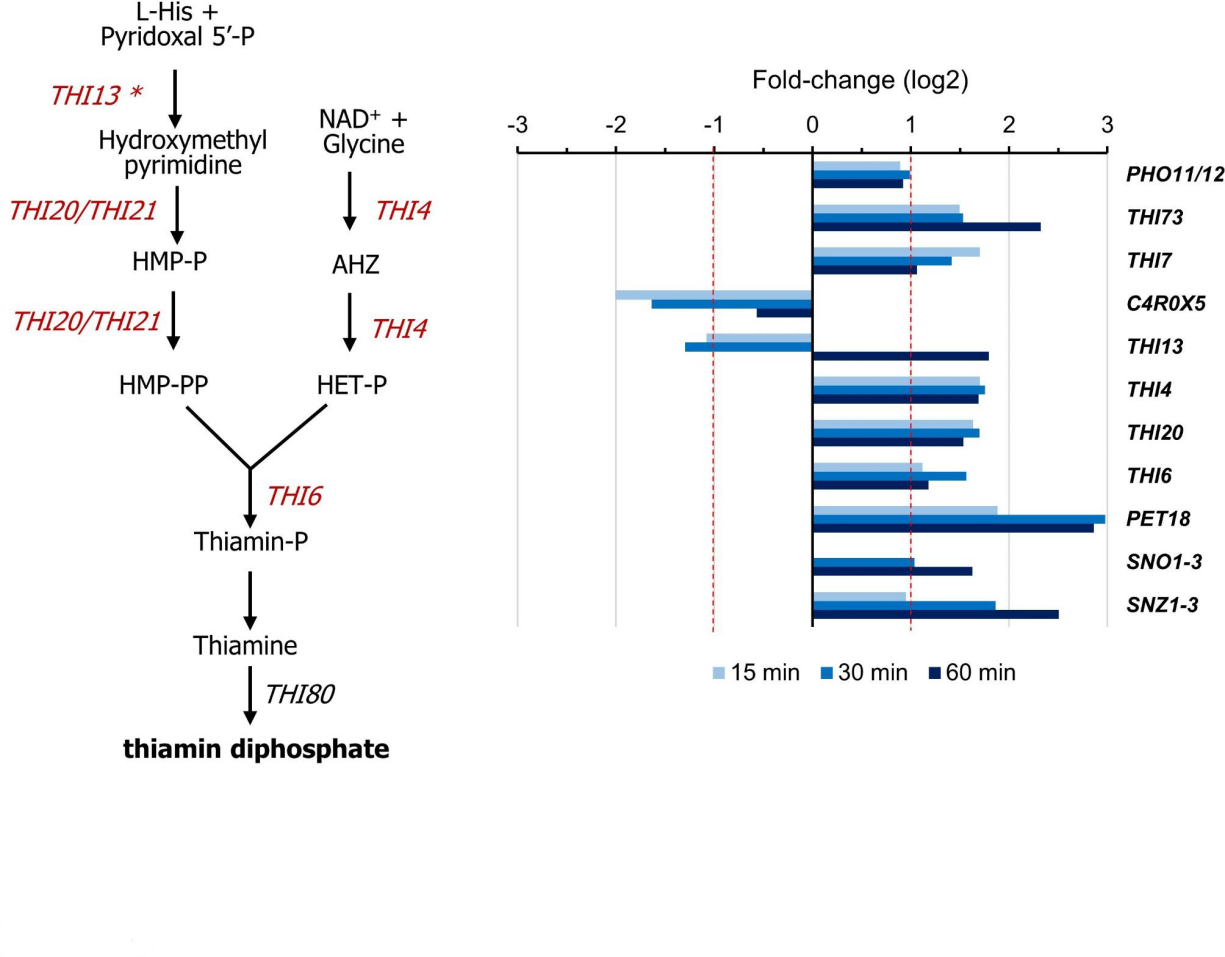
Transcriptional effects on the thiamine biosynthetic pathway. Data corresponds to cells gown on glycerol and shifted to pH 8.2. Induced genes are in red. The asterisk (*) in *THI13* indicates that this gene is repressed at time 15 and 30 min, but induced at 60 min

Alkalinization triggers the induction of genes involved in the uptake and biosynthesis of thiamine, a response very similar to that observed upon thiamine starvation. As shown in Fig. 6, an increased expression of genes encoding C4QZN1 (a Pho11/12 phosphatase, able to hydrolyze thiamin phosphates in the periplasmic space), two plasma membrane thiamin transporters (C4QWN5/Thi73 and C4R561/Thi7), and several enzymes of the thiamine biosynthetic pathway, such as Thi4, Thi20 and Thi6 was observed. Curiously, Thi13, which catalyzes the first step in the pathway, was initially repressed and potently induced later. The gene encoding C4R0 X 5, a putative mitochondrial thiamine pyrophosphate carrier, was strongly repressed. At least three genes encoding proteins known to be induced in *S. cerevisiae* upon thiamine starvation, such as C4QZX3 (Pet18), C4QYB3 (Sno1-3), and C4QYB4 (Snz1-3) were also clearly induced by high pH. The latter two genes encode activities required for the synthesis of pyridoxal 5’-phosphate, the substrate for Thi13.

**Fig. 7:**
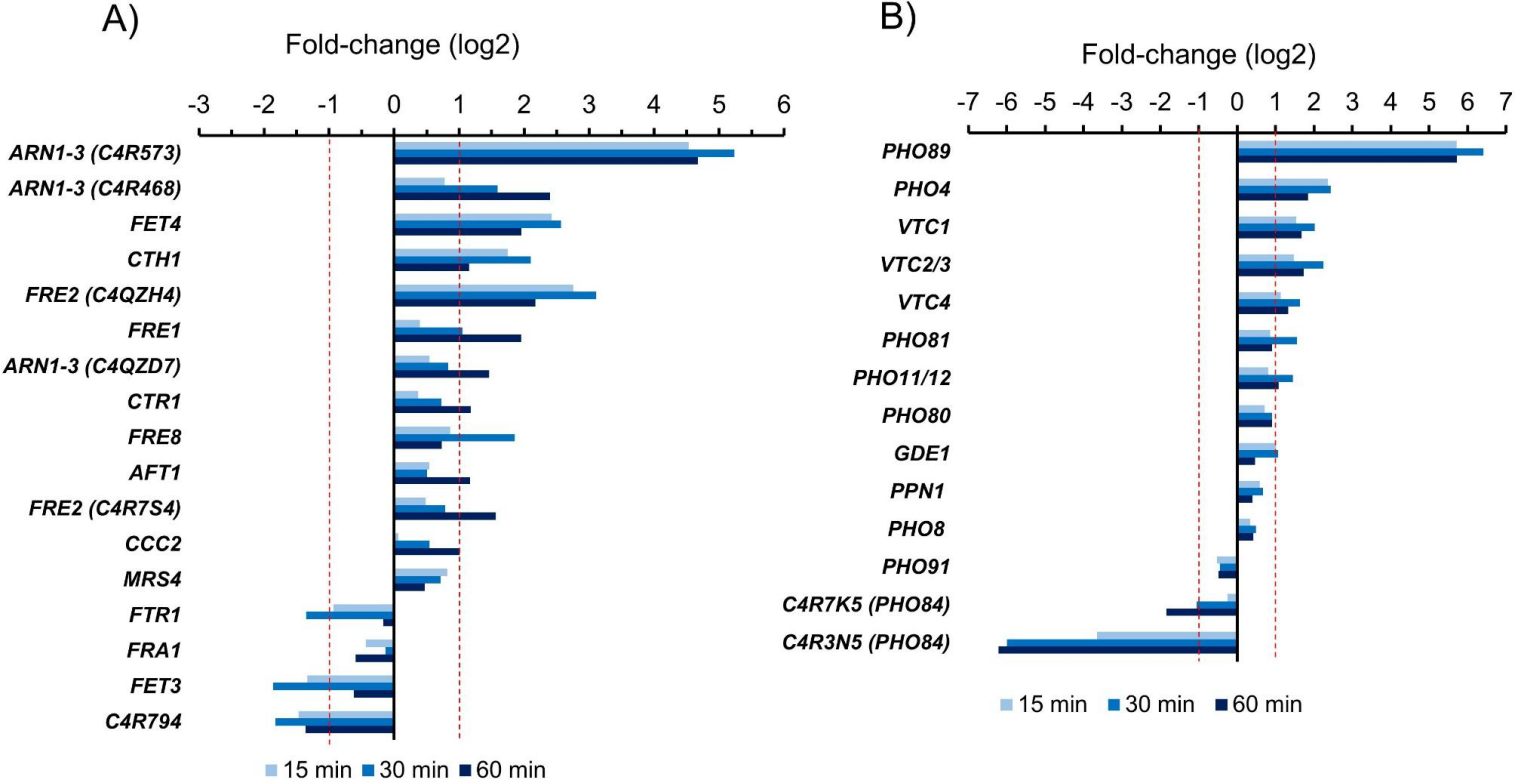
Transcriptional effects on the iron and copper (A) and phosphate uptake and utilization (B) pathways. In both cases, cells were grown on glycerol and shifted to pH 8.0. See main text for details

As shown in Fig. 7A, the expression of genes involved in copper and iron metabolism was potently altered. Among them, at least three different Arn1-3 like siderophore transporters (C4R573, C4R468, and C4QZD7), two putative members of the ferric and cupric reductase system (Fre1 and Fre2), the low-affinity Fe(II) transporter Fet4, and the plasma membrane high-affinity copper transporter Ctr1 (C4R733) were induced. The gene encoding the high-affinity zinc transporter of the plasma membrane (C4R4S9, Ztr1) was among the most potently induced genes (not shown). Interestingly, two important components of the iron uptake system, Fet3 (C4R1S1) and Ftr1 (C4QX98), were clearly repressed. Fet3 is the plasma membrane Ferro-O_2_-oxidoreductase that oxidizes Fe^2+^ to Fe^3+^ for subsequent uptake by the transmembrane permease Ftr1.To note, the gene encoding C4R794, a protein very closely related to *S. cerevisiae* Fre2 and Fre3, was also markedly repressed.

Phosphate metabolism appears also affected by alkalinization of the medium Fig. 7B. This included genes encoding components of the vacuolar polyphosphate biosynthetic machinery (Vtc1, Vtc2/3, Vtc4), the putative transcription factor Pho4 (C4QZW1) and the Na^+^/Pi cotransporter Pho89 (C4R021), which was one of the genes most potently induced. In contrast, we found two genes encoding putative plasma membrane phosphate transporters (C4R3N5 and CAR7K5), equivalent to the *S. cerevisiae* Pho84 H^+^/Pi high affinity transporter, that were repressed by alkaline pH. The one encoding protein C4R3N5 was among the most strongly repressed genes.

Taken together, these results indicate that the alkalinization of the medium triggers a transcriptional response that affects the metabolism of arginine and sulfur-containing amino acids, thiamine, as well as the homeostasis of copper, iron and phosphate.

**Alkalinization causes a widespread transcriptional repression of respiratory metabolism**.

**Fig. 8:**
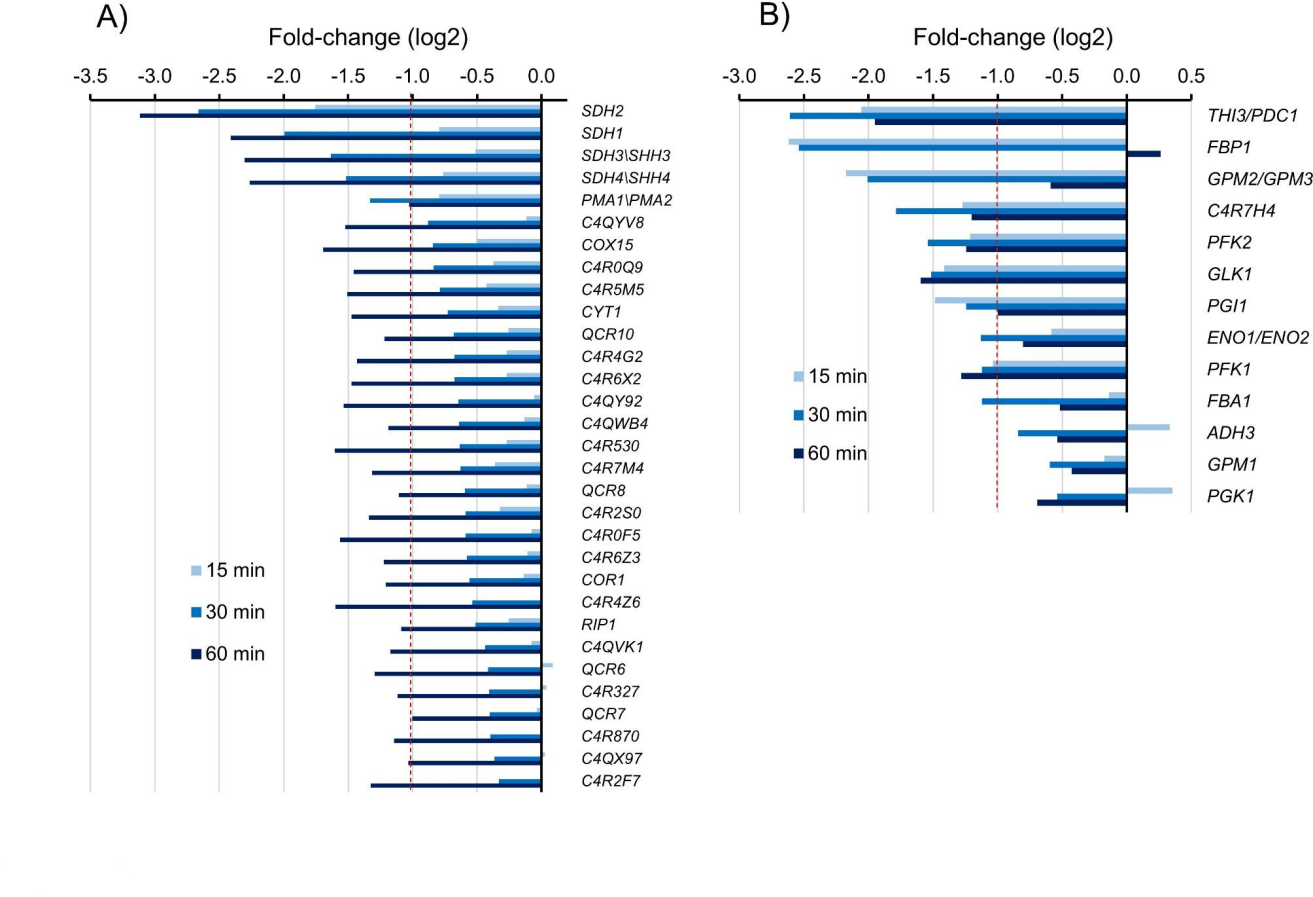
Repression of oxidative phosphorylation (A) and glycolysis/gluconeogenesis related (B) genes. Data corresponds to glycerol cultures shifted to pH 8.2. See main text for details

GO analysis of genes showing a down-regulation pattern highlighted many genes related to oxidative phosphorylation (Figs. 2 and 3). As shown in Fig. 8A, changes increased with time and were more intense at the latest time tested (60 min). Down-regulated genes included widespread components of the electronic transport chain, involved in generation of complex II (*SDH1*, *SDH2*, *SDH3* and *SDH4*), complex III (*CYT1*, *COR1*, *RIP1*, *QCR6*, *QCR7*, *QCR8*, and *QCR10*), and complex IV (COX15), as well as several other related but less well characterized proteins. In addition, a decrease in the mRNA levels of many genes encoding glycolytic and gluconeogenic enzymes was observed (Fig. 8B). For instance, all genes encoding the subunits of phosphofructokinase-1 (PFK1), a key regulator of glycolysis, were repressed, including the one coding for C4R7H4, a γ PFK1 subunit that is not found in other fungi. We noted that *ADH3*, *GPM1* and *PGK1*, which suffered a weak repression in glycerol cultures, were more potently repressed when grown on glucose, decreasing below the log2 <= -1 threshold.

### Characterization of the response of selected genes by translational promoter fusions

**Fig. 9:**
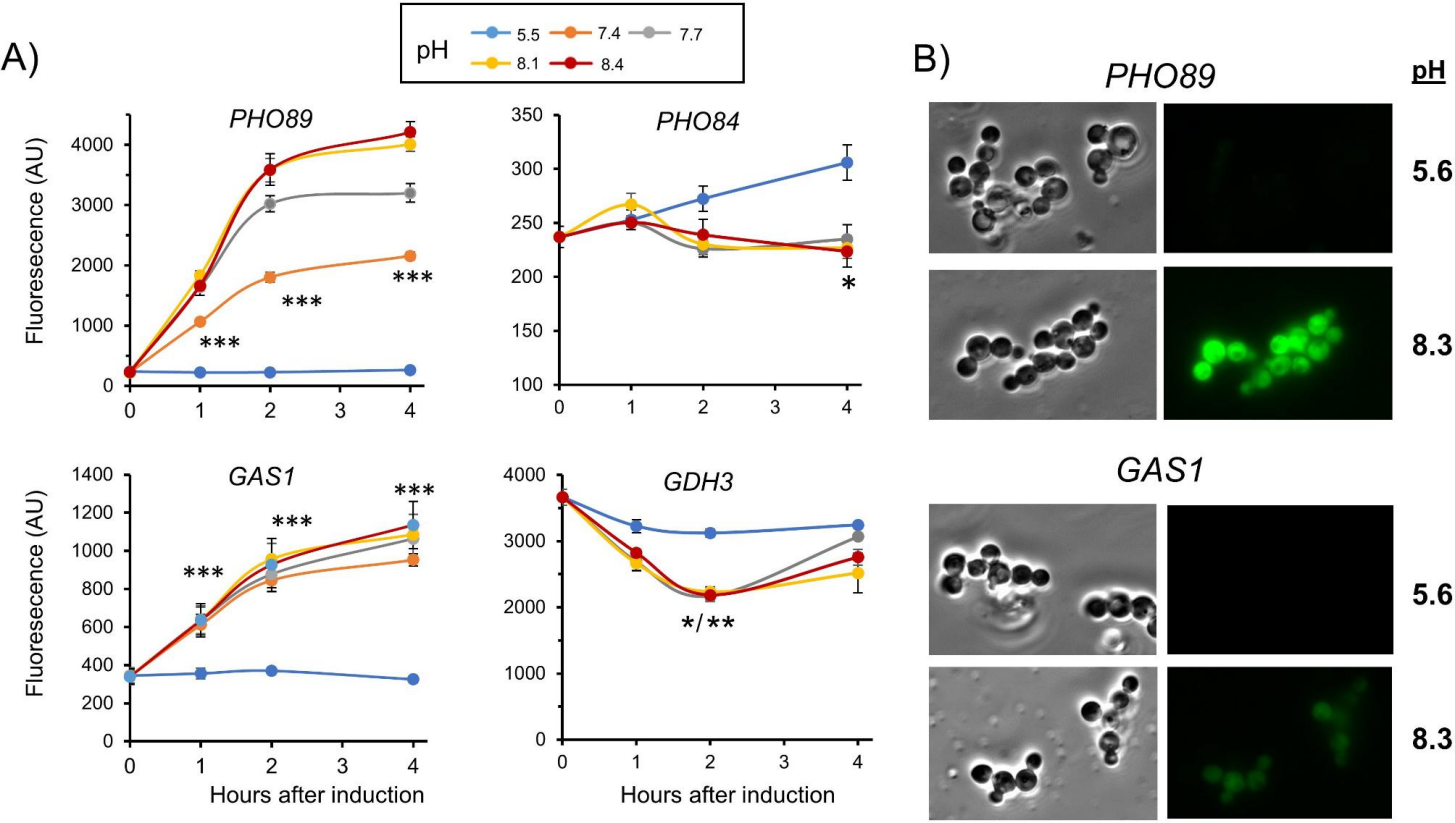
Analysis of pH dependent promoter response of selected genes by translational reporters. (**A**) The indicated GFP-derived reporters were introduced in *K. phaffii* X-33 cells and cultures were shifted to the different pHs by addition of KOH. Samples were taken at the indicated times and processed for flow cytometry analysis. Data are mean ± SEM from 3 to 5 experiments. *, p < 0.05; **, p < 0.01; ***, p < 0.001 determined by paired Student’s t-test. (**B**) Cultures bearing *PHO89*-GFP and *GAS1*-GFP constructs were monitored by fluorescence microscopy after growing at normal pH (5.6) or alkaline pH (8.3) for 4 h

To further test the transcriptional response to alkaline pH determined by RNA-Seq we selected two strongly induced genes (*PHO89* and *GAS1*) and two repressed ones (C4R3N5, corresponding to one of the two likely *PHO84*, and *GDH3*), for GFP-fused promoter reporter assays (Additional File 1). As mentioned above, *PHO89* and *PHO84* encode putative Na^+^/Pi and H^+^/Pi plasma membrane high affinity transporters, respectively, and were selected because their unexpected opposite behavior (see Discussion). *GAS1* encodes a putative β-1,3-glucanosyltransferase, required for cell wall assembly, and *GDH3* codes for a putative NADP^+^-dependent glutamate dehydrogenase. As shown in Fig. 9A, *PHO89* and *GAS1* reporters confirmed the strong activation of both promoters by alkaline pH. We noted that they presented different pH sensitivity, since *GAS1* was almost maximally induced already at the lowest pH tested (7.4), whereas *PHO89* displayed a marked dose-dependent response. The activation of *PHO89* and *GAS1* could be also easily monitored by fluorescence microscopy (Fig. 9B). Analysis of *PHO84* and *GDH3* confirmed the repression of these promoters, although the changes were less potent than that detected by RNA-Seq (compare with Additional file 1) and showed different kinetic profile.

**Fig. 10:**
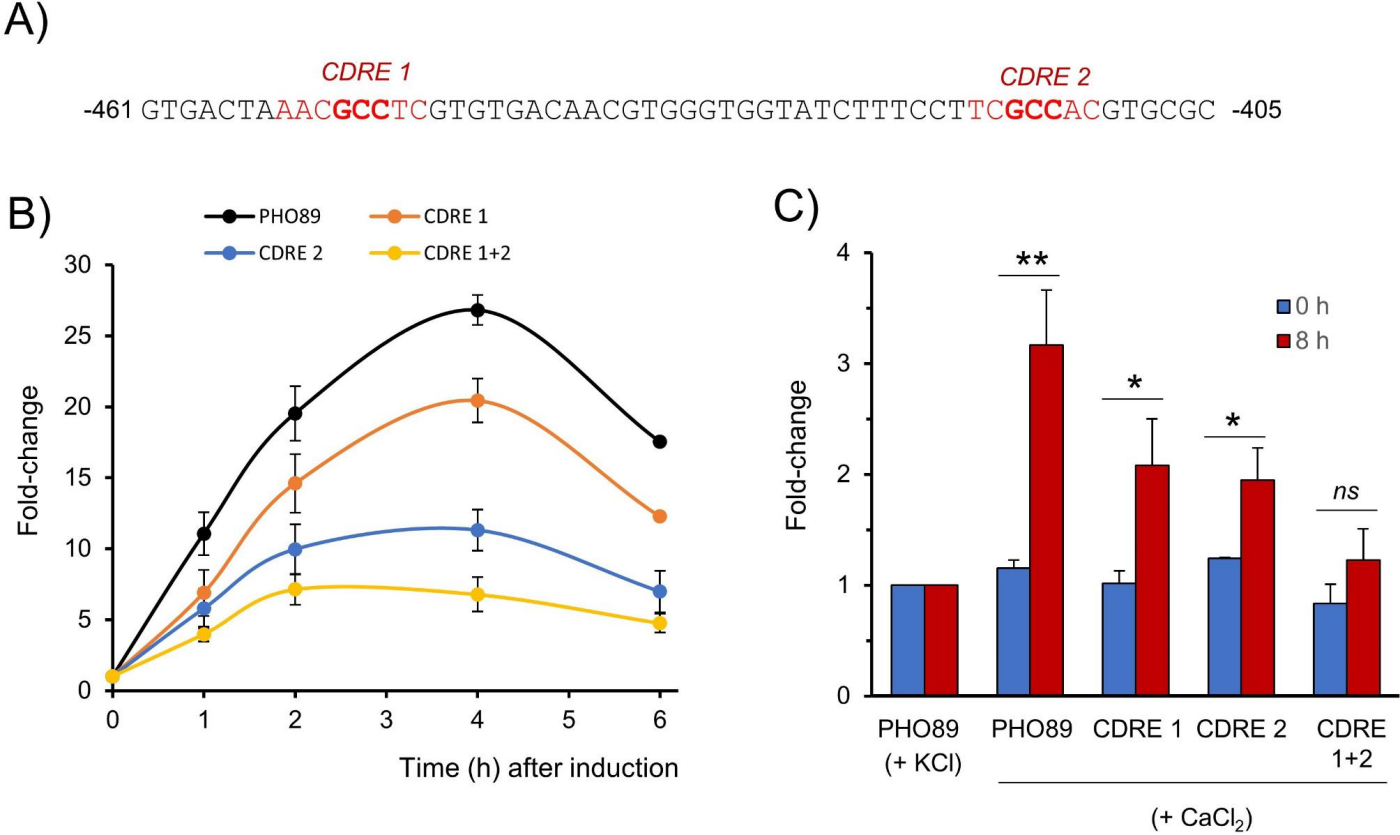
Putative CDRE elements are crucial for pH-dependent induction of *PHO89* expression. (**A**) Putative CDRE elements were identified in the *PHO89* promoter (CDRE 1 and CDRE 2). (**B**) The constant GCC core was mutated individually or in combination (CDRE 1 + 2) and the response of cells bearing *PHO89*-GFP reporters carrying such mutations was compared by flow cytometry with that of the native version (denoted as PHO89). Data is represented as the mean ± SEM of the fold-change in comparison with unstressed cells from 4 experiments. (**C**) Cells bearing the reporters indicated above were incubated for 8 h with 0.6 M KCl or CaCl_2_ and samples taken at t = 0 and 8 h for flow cytometry analysis. Values were compared with cells incubated with KCl to avoid interference of possible osmotic effects. Data is the mean ± SEM of the fold-change from 3 or 4 experiments. *, *p* < 0.05; **, *p* < 0.01; ns, not significant (p > 0.05)

In an earlier report it was shown that, in *S. cerevisiae*, the induction of *PHO89* by alkaline pH is mainly mediated by the calcineurin-Crz1 pathway in response to the immediate influx of calcium into the cell [7]. We sought to investigate if this would be the case in *K. phaffii*. Computational analysis of the *PHO89* promoter in *K. phaffii* revealed diverse putative regulatory sites, including a possible Pho4 site, but also two putative CDREs (Calcineurin Dependent Response Element), that we named CDRE 1 and CDRE 2 (Fig. 10A). To test the possible functional role of these sites, we mutagenized both of them at the crucial GCC core either separately or in combination. As shown in Fig. 10B, mutation of either CDRE site decreased the response of the reporter to alkalinization, although the effect of the mutation of CDRE 2 was more prominent than that of CDRE 1. Removal of both sites further decreased the promoter response to less than 20% of the observed for native version. These results indicate that both sites are functional (although not equally relevant) and that in *K. phaffii* the *PHO89* gene is likely subjected to transcriptional regulation by the calcium-calcineurin pathway. Further support for this notion was obtained by incubating the cells with CaCl_2_ and monitoring the activity of the native and mutated promoters (Fig. 10C). We observed that the *PHO89* reporter was responsive only when rather high concentrations of CaCl_2_ (≥ 0.6 M) were added to the medium and after a relatively long period of incubation (> 4 h). In any case, the induction caused by external calcium was partially abolished by mutation of either CDRE 1 or CDRE 2, and fully eliminated when both sites were altered, thus supporting the calcium-mediated nature of *PHO89* induction in response of alkaline pH.

### Expression levels achieved by pH-regulatable alkaline promoters are in the range of those of constitutive promoters commonly used for expression of heterologous proteins

**Fig. 11:**
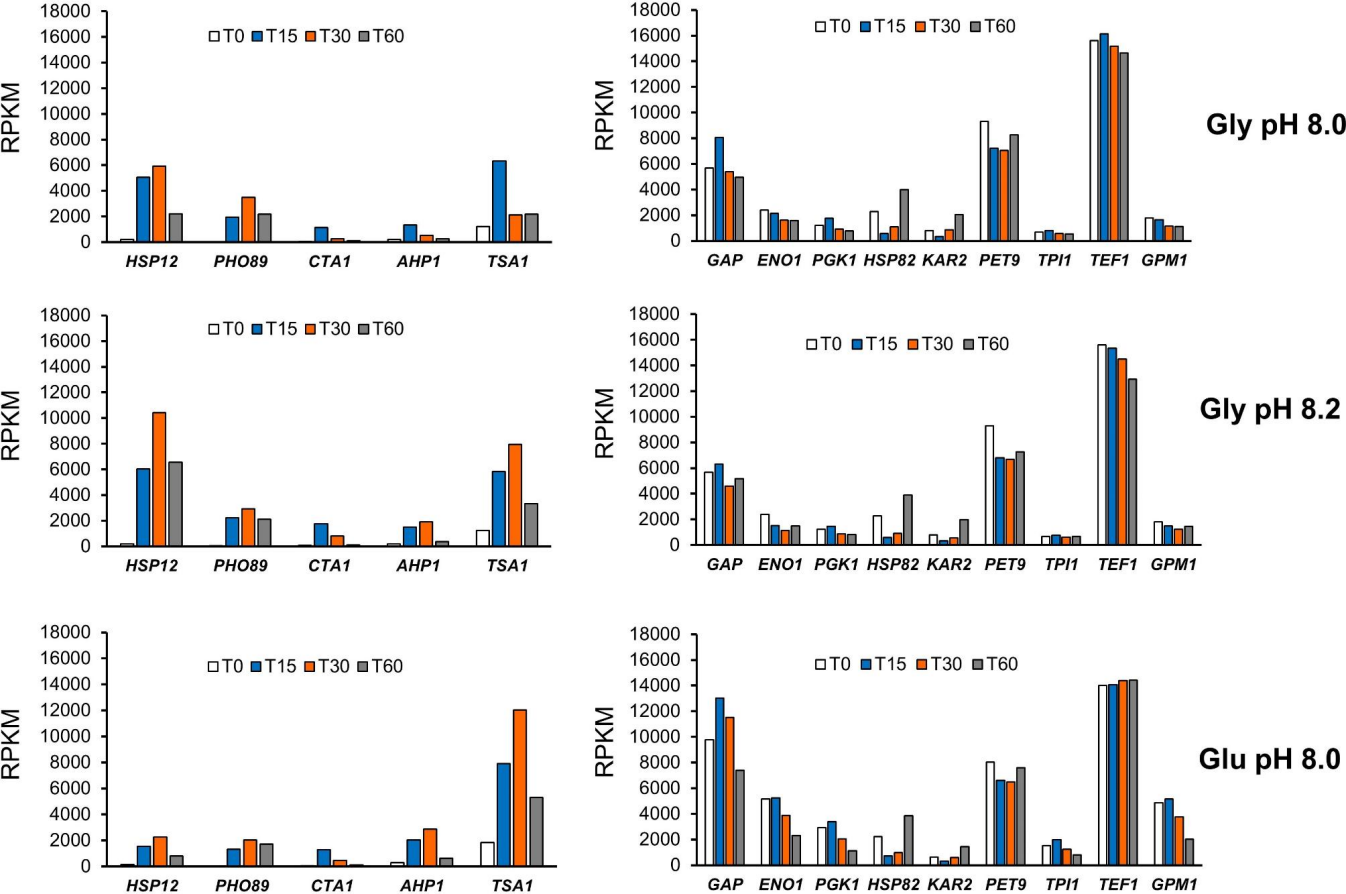
Relative expression of selected alkaline pH-inducible genes and constitutively expressed genes whose promoters are widely used for heterologous protein expression. The RPKM (reads per kilobase per million mapped reads) were calculated for each gene and condition in our dataset and plotted for five selected alkaline-inducible genes and nine constitutive genes (the latter selected from Table 1 in reference [25]). The scale of the Y axes has been kept the same in all graphs to facilitate comparison

To evaluate the usefulness of the newly discovered alkaline pH-regulatable promoters for industrial application we sought to assess the amount of mRNA generated in comparison with that of genes whose expression is driven from well-known constitutive promoters widely used for heterologous protein production [25]. To this end we calculated the RPKM (reads per kilobase per million mapped reads) values for each condition, which allow comparing gene expression values within a sample. As shown in Fig. 11, expression of *HSP12* and *TSA1* nearly matched that of GAP, and it was from 65 to 75% of that of *TEF1*, which was the second most potently transcribed gene in our dataset. *PHO89* was the most intensely induced gene by alkalinization (nearly 90-fold, see Additional File 1, Supp. Figure 3 and Additional File 2), and its expression level in cells grown on glycerol was higher than that of *PGK1* or *TPI1* in cells growing on either carbon sources. Therefore, the mRNA levels attained upon induction from alkaline pH-regulatable promoters are high enough to suggest that they could be used to develop novel platforms for heterologous protein expression in *K. phaffii*.

## Discussion

Work in the last twenty years has shown that environmental alkalinization results in robust transcriptional remodeling that affects hundreds of genes not only in *S. cerevisiae* [7, 12, 13, 26–28], but also in other fungi such as *C. albicans* [29], *Aspergillus nidulans* [29, 30] or the mycotrophic fungus *Trichoderma virens* [31]. Data presented in this report indicates that this is also the case for the methylotrophic yeast *K. phaffii*, for which we detect over 700 genes up- or down-regulated. This was somewhat surprising, since even at the higher pH tested (8.2) the impact on growth rate was relatively mild (see Additional File 1, Supp. Figure 4) in comparison with *S. cerevisiae*, which hardly grows at pH 8.2 or above. We also observe that the overall response is quite similar irrespectively of the carbon source used (Fig. 1). On the other hand, increasing the intensity of the stress (from pH 8.0 to 8.2) tends to delay the transcriptional response (Figs. 1B and C and 3). This is reminiscent of what has been reported in response to osmotic stress in *S. cerevisiae* [32–34], likely as a result of a more prolonged adaptation phase until effective transcription occurs.

We show that alkaline pH leads to the development of a situation of oxidative stress in *K. phaffii*. This was previously observed for *S. cerevisiae* [7, 12, 35], although it was not reported in the case of *C. albicans* [29]. We also detect a marked increase in a subset of genes required for assimilation of iron and copper. The induction of this kind of genes upon alkalinization has been observed in *S. cerevisiae* and *C. albicans* [12, 28, 29] and the availability of these metals at alkaline pH has been shown to be a limiting factor for growth in *S. cerevisiae* and *S. pombe* [35, 36]. However, the specific expression profile of iron-related genes in *K. phaffii* is somewhat particular. Indeed, many genes belonging to the non-reductive uptake system, encoding siderophore transporters as well as the oxygen-independent low-affinity plasma membrane iron and copper transporter Fet4, were induced. In contrast, the genes encoding the copper-dependent high-affinity iron import complex Fet3/Ftr1, as well as a putative homolog of Fre3, one of the cell surface metalloreductases that cooperates with Fet3/Ftr1 [see [37] and references therein] were clearly repressed. Whereas induction of genes relevant for the non-reductive pathway could be mainly attributed to the decreased bioavailability of iron, derived from the low solubility of this metal (particularly as Fe^+ 3^) at neutral and alkaline pH [38], the decrease in mRNA of *FET3* and *FTR1* could be due to the concurrent condition of oxidative stress. In this regard, it has been shown that in *S. cerevisiae* under oxidative stress caused by *ter*-butyl hydroperoxide [39, 40] or arsenate [41] *FTR1* and *FET3* mRNA levels are maintained low by transcript destabilization through the general 5’–3’ mRNA decay pathway. This response would allow redirecting iron assimilation through the non-reductive pathway, thus diminishing oxidative damage derived from ferrous ions import through the Ftr1/Fet3 complexes [38]. In contrast, putative *FTR1* and *FET3* homologs are found induced upon environmental alkalinization in *C. albicans* [29].

Related to iron metabolism, we also observe induction of the gene encoding C4R7J6 (named here *CTH1*), which is the closest relative to *S. cerevisiae* Cth1 and Cth2 (Tis11) proteins. It is known that, in *S. cerevisiae*, Cth2 acts as a mRNA-binding protein that, by posttranscriptional inhibition, restricts the expression of genes coding for iron-containing proteins or that participate in iron-consuming processes [see [37] and references therein]. These include components of the mitochondrial electron transport chain or the tricarboxylic acid (TCA) cycle. Indeed, we observe a marked decrease in the genes encoding proteins containing iron-sulfur clusters (Figs. 2 and 3), such as *SDH2*, *LEU1*, *LYS4*, *ACO1*, *ACO2*, *GLT1*, and *GRX3*, among others. Repression of iron-sulfur containing genes (*SDH2*, *GLT1*, *COX15*, among others) can be also observed in *C. albicans* [29]. In addition, we detect an overall repression of genes involved in oxidative phosphorylation, mostly encoding components of the electron transport chain, as well as additional elements of the TCA cycle, such as isocitrate dehydrogenase (C4R142) or the Kgd2 subunit of α-ketoglutarate dehydrogenase, which possibly contribute to the smaller growth rate of the culture in the first few hours (Additional File 1, Supp. Figure 4). Therefore, it is likely that most of the changes described above would be the result of the combination of iron unavailability and oxidative stress. It is worth noting that in the halotolerant yeast *Debaryomyces hansenii*, which is mainly a respiratory yeast, a down-regulation of genes involved in glycolysis and oxidative phosphorylation was also reported in response to alkalinization of the medium [42].

Diverse metabolic pathways are upregulated in *K. phaffii* by high external pH, including the biosynthesis of arginine and sulfur-containing amino acids, as well as the biosynthesis of thiamine. The behavior of arginine metabolism in response to alkalinization appears to be species dependent. Thus, in *S. cerevisiae*, genes involved in arginine biosynthesis are consistently up-regulated [7, 10, 13, 26], although *CAR1* and *CAR2*, involved in arginine utilization, appear also up-regulated. Up-regulation of arginine biosynthetic genes is also observed in *A. nidulans*, accompanied by a strong down-regulation of AN8279, encoding a homolog of the plasma-membrane arginine transporters *CAN1* and *GAP1* [30]. In sharp contrast, the expression pattern in *C. albicans* in response to alkaline pH is the opposite: biosynthetic genes are repressed, and degradative ones are induced [29]. Concerning methionine metabolism, alkaline pH triggers the induction of the biosynthetic pathway in *S. cerevisiae*, including genes (*MUP1*, *MUP2*) involved in the uptake of the amino acid [7, 10, 26]. In contrast, the effect of alkalinization on this pathway seems very limited in *C. albicans* and none in *A. nidulans* [29, 30]. It is worth noting that, in addition to environmental alkalinization, a concomitant induction of genes involved in biosynthesis of arginine and methionine has been observed by transcriptomic and/or metabolomic analyses in *S. cerevisiae* cells subjected to different forms of stress, such as heat stress [43, 44], arsenate [45] and sulfur dioxide [46] treatments, or potassium starvation [47]. It is suggestive that all these stress conditions are accompanied by a situation of oxidative stress, as we show here for *K. phaffii* under alkaline stress. Therefore, it is tempting to speculate that the concerted induction of arginine and methionine biosynthetic genes would be a response to oxidative stress elicited by extracellular alkalinization. This scenario is supported by the previous observation that the beneficial effect on ethanol tolerance in *S. cerevisiae* derived from arginine overproduction was, at least in part, due to decreased ROS production [48]. In addition, deletion of diverse genes encoding methionine or arginine biosynthetic genes was shown to strongly decrease tolerance to sulfur dioxide stress [46]. Interestingly, in *C. albicans*, where arginine biosynthesis is repressed and methionine biosynthetic genes are barely affected, exposure to alkaline pH does not generate a strong transcriptional oxidative stress response and, in fact, diverse genes induced in *K. phaffii* are even repressed (i.e., *SOD2*, *CAT1*, *TTR1, AHP1* or *HSP21*).

The increased expression of thiamine biosynthetic genes observed here is intriguing because this is a very expensive process from the point of view of energy and it is mainly regulated at the transcriptional level [see [49] and references therein]. Indeed, their expression seems largely unchanged in alkali-treated cultures of *S. cerevisiae*, *C. albicans* or *A. nidulans*. Still, the emergence of these genes as induced in *K. phaffii* could be related to oxidative stress. For instance, some of them (i.e. *THI13, THI4, THI20, or THI6*) were identified as activated in response of heat shock [44] and, in addition, an increase in both the transcript levels and the enzymatic activity was observed for Thi4 and Thi6 in yeast subjected to oxidative stress by hydrogen peroxide [50].

We have found that diverse genes involved in the response to phosphate scarcity are induced by alkalinization in *K. phaffii*. Among them, the Na^+^/Pi transporter *PHO89* was among the most potently induced genes. Induction of *PHO89* by low phosphate conditions in *K. phaffii* was reported earlier [51] and these same authors highlighted the presence of two putative Pho4 binding sites (CACGTG/T) at positions − 583 and − 414 from the initiating ATG (albeit these sites were not further characterized). We reported long ago [7, 12] that, in *S. cerevisiae*, alkaline pH induction of *PHO89* was largely dependent on the activation of the calcineurin pathway and the Crz1 transcription factor. Our data demonstrates that in *K. phaffii* the *PHO89* promoter contains a tandem of two putative Crz1 binding sites, located at positions − 405/-461 that, working together, are almost fully responsible for the alkaline pH response of this gene. These sites are also responsible for the induction of *PHO89* in response to extracellular calcium. It must be noted that *K. phaffii* seems rather refractory to the internalization of extracellular calcium under standard growth conditions, since very high amounts of the cation must be added to elicit *PHO89* induction. In any case, our data suggest that environmental alkalinization promotes the entry of extracellular calcium in *K. phaffii* which, through activation of calcineurin and the Crz1 transcription factor, would activate *PHO89* expression. In addition to *S. cerevisiae* [7, 12], the sensitivity of *PHO89* to alkaline pH has been also documented for *Candida albicans* [8, 29] and *Aspergillus nidulans* [30]. Therefore, the regulation of this Na^+^/Pi antiporter gene by high pH through the calcium/calcineurin signaling seems a widespread feature in fungi, pointing out to a conserved mechanism.

It is worth noting that, together with *PHO89*, previous work in *S. cerevisiae* [12, 28] and *C. albicans* [29] has identified also the gene encoding the H^+^/Pi cotransporter *PHO84* as upregulated in response to high pH. *K. phaffii* contains two genes encoding Pho84-like proteins (C4R3N5 and C4R7K5) but, in this case, we observe a strong decrease in their mRNA levels, particularly in the case of C4R3N5. Indeed, analysis of the gene promoter by using a GFP reporter confirmed a moderate and relatively slow decrease in activity (Fig. 9). However, it is difficult to justify the very fast and potent decrease in *PHO84* mRNA levels solely based on promoter repression. One possible explanation would be a rapid destabilization of *PHO84* mRNA. In fact, a fast mRNA destabilization upon environmental alkalinization was shown to be a general trait for genes showing a reduction in their mRNA levels in *S. cerevisiae* [27]. While the downregulation of putative Pho84-encoding genes in response to high pH observed here might appear surprising, it is worth mentioning that the recent analysis of the transcriptome of *A. nidulans* in response to alkalinization [30] reveals a clear down-regulation (∼4-fold) of ANIA_01612, which codes for the closest relative to *S. cerevisiae* Pho84. Therefore, it seems that whereas induction of *PHO89* by high pH appears a common feature in fungi, the specific response of Pho84-encoding genes appears very much determined by the fungal species.

## Conclusions

In conclusion, our work has uncovered a vast array of genes that are sensitive to a moderate alkalinization in the yeast *K. phaffii*. Due to the interest of this organism for heterologous protein expression and that (1) modulation of the pH of the medium is a very easy and economical procedure, and (2) the expression level achieved in some cases is comparable to that of well-known strong constitutive promoters, some of these genes may provide novel promoters useful for this purpose. Examples would be *HSP12*, *TSA1* or even *PHO89*, a gene potently induced by alkaline pH and whose performance in response to phosphate starvation was previously found to be comparable to that of well-known strong *K. phaffii* promoters, such as of the genes encoding translation elongation factor 1α and glyceraldehyde-3-phosphate dehydrogenase [51]. Understanding how *K. phaffii* signals and adapts to moderate alkalinization will provide information to further refine the response of alkaline pH-regulatable promoters to enhance their suitability for protein production at the industrial level.

## Materials and methods

### Yeast strains and growth conditions

*K. phaffii* X-33 was grown at 28 °C in YP medium (10 g/L yeast extract, 20 g/L peptone) supplemented with 20 g/L of glycerol (YPGly) or glucose (YPD) with continuous shaking (230 rpm/min). The medium contained 200 µg/ml of hygromycin B for selection of transformants. Growth data under different pH conditions were obtained by monitoring the OD_600_ in a Bioscreen C apparatus (Thermo Fisher) as described in [52].

### DNA techniques

*Escherichia coli* (strain DH5α) was used as standard plasmid DNA host and was grown in LB medium at 37 °C supplemented with 100 µg/ml hygromycin B when needed for plasmid selection. Transformation of *E. coli*, and standard recombinant DNA techniques were performed as described [53]. Yeast cells were transformed by electroporation following the procedure described in [54].

### Construction of gene reporters

Gene reporters were made by translational fusion to eGFP and subsequent integration in the *K. phaffii* genome. To this end, we amplified by PCR from genomic DNA obtained from strain X-33, using Q5 DNA polymerase (New England Biolabs), the following genomic regions (from the initiating ATG codon): -772/+11 from *PHO89* (PAS_chr2-1_0235); -839/+6 from *GAS1* (PAS_chr1-3_0227), -981/+21 from *PHO84* (PAS_chr3_0141), and − 752/+30 from *GDH3* (PAS_chr1-1_0107). The first three fragments were digested with HindIII and KpnI, whereas the *GDH3* region was digested with BstBI and KpnI. All fragments were ligated into the corresponding sites of plasmid pAHYB–GFP [55] and the mixtures used to transform *E. coli* cells. Positive clones were selected in LB plates containing hygromycin B. The inserts from selected clones were sequenced to ensure the absence of unwanted mutations.

Mutation of the putative CDRE consensus sequences in the *PHO89* promoter was done as follows. Mutation of CDRE-1 (nt -455/-446) was made by two-step PCR, using the pAHYB-PHO89 reporter as template. In the first step, two fragments were amplified using primer pairs Pp_Pho89_Fw / PHO89-CDRE-1-Rev, and PHO89-CDRE-1-Fw / Pp_Pho89_Rev (348 and 492 bp, respectively). Both amplification fragments were diluted and re-amplified with oligos Pp_Pho89_Fw and Pp_Pho89_Rev. For CDRE-2, the strategy was the same but using primers PHO89-CDRE-2-Fw and PHO89-CDRE-2-Rev in the first step reactions, to yield fragments of 381 and 454 nt, respectively. Upon digestion with HindIII and KpnI and cloning into pAHYB-GFP, the resulting constructs were denominated pPHO89-CDRE-1 and pPHO89-CDRE-2, respectively. The double mutation was constructed in parallel by mutating CDRE-2 using pPHO89-CDRE-1 as template, and mutating CDRE-1 using pPHO89-CDRE-2 as amplification source, yielding in both cases plasmid pPHO89-CDRE-1 + 2. In all cases, plasmids were linearized within the *AOX1* promoter with PmeI, and 100 ng of DNA used to electroporate X-33 cells. Transformants were recovered in YPD plates containing hygromycin (200 µg/ml). The correct insertion of the plasmid into the chromosomal *AOX1* locus was confirmed by PCR. Oligonucleotides used in this work and details on their use are documented in Additional file 3. Promoter analysis was performed with the RSAT platform [56].

### RNA purification and RNA-Seq procedures

For the analysis of the transcriptional response of *K. phaffii* to alkalinization of the medium cells were grown overnight on YPD or YPGly medium, and then cultures were diluted in 200 ml of fresh medium at OD_600_ = 0.2 (∼ 10^7^ cells/ml) and grown until an OD_600_ around 0.6. Then two 100 ml-aliquots were centrifuged (5 min at 1000xg). One aliquot was resuspended in the same volume of either fresh YPD or YPGly medium plus 50 mM TAPS pH 5.5 (control cells) or in YPD or YPGly supplemented with 50 mM TAPS adjusted to pH 8.0 or 8.2 (stressed cells). Samples (15 ml) were taken immediately after resuspension in medium at pH 5.5 (time = 0) and, for each stressed culture, after 15, 30 and 60 min. In all cases, samples were placed on ice and immediately centrifuged at 4 °C. Total RNA was extracted with the Ribo Pure™-Yeast kit (Ambion™) following the manufacturer’s instructions. The quality was assessed by capillary electrophoresis in a Bioanalyzer equipment and RNA amounts quantified by measuring the A_260_ in a Nanodrop ND-1000 Spectrophotometer.

mRNA was purified from total RNA using poly-T oligo-attached magnetic beads. After fragmentation, the first strand cDNA was synthesized using random hexamer primers, followed by the second strand cDNA synthesis using dTTP (non-directional library). The library was checked by Qubit™ assay (Thermo Fisher Scientific) and real-time PCR for quantification and size distribution monitored with a Bioanalyzer 2100 equipment (Agilent). Quantified libraries were pooled and sequenced on an Illumina NovaSeq 6000 platform by Novagene Co. Ltd. An average of 22.8 million of single reads per sample were used for subsequent analysis (98.7% were effective reads and 94.49% were Q30 or higher on average). Two biological replicas were done for each condition, except for pH 8.0 at 15 min, for which data from four biological replicas was obtained. DNA sequence data was deposited at NCBI’s Gene Expression Omnibus (GEO) and can be retrieved under study GSE224427.

### Analysis of RNA-Seq data

Mapping of fastq files to generate SAM files was carried out with the Bowtie2 software [57] in local / sensitive mode against the GCA_000027005.1 assembly (https://www.ncbi.nlm.nih.gov/assembly/GCA_000027005.1/), yielding 98.4–99.2% mapped reads. The SeqMonk software v1.48.0 (https://www.bioinformatics.babraham.ac.uk/projects/seqmonk/) was employed to analyze the SAM files. Mapped reads were counted using the RNA-Seq pipeline on mRNA features and differentially expressed genes with respect to time zero were identified using the built-in DESeq2 R package, selecting the multiple testing correction option [58]. Genes with a p-value < 0.01 (FDR < 0.05) and a log2 fold-change <-1 or > 1 were selected for further analysis. Functional annotations were obtained from NCBI’s “Feature Table” of the GCA_000027005.1 assembly and enriched with data retrieved from Uniprot (Proteome ID UP000000314). In addition, to gain for further information, a Blastp search against the proteomes of *Saccharomyces cerevisiae*, *Schizosaccharomyces pombe*, *Candida albicans*, *Aspergillus nidulans* and *Cryptococcus neoformans* proteomes was carried out, setting a p-value threshold of 10^− 30^. Gene Ontology analyses for functional profiling were done with the g.Profiler server (https://biit.cs.ut.ee/gprofiler/gost) using the *K. phaffii* GS115 data from the Ensemble Genomes fungi database.

### Evaluation of reporter expression

For investigation of the response of reporters to pH changes, o/n cultures were diluted in YPD to OD_600_ = 0.3–0.4 and grown until OD_600_ = 0.6. Cultures were split into aliquots (8–12 ml), and the appropriate volume of 1 M KOH added to achieve a final concentration of 20–35 mM (pH 7.4 to 8.4). Samples (0.875 ml) were taken at different times and cells were fixed by mixing with 50 µl of formaldehyde (37.5% solution) for 5 min at room temperature, washed twice with the same volume of cold PBS and finally resuspended in 200–300 µl of PBS. Fluorescence of the samples was analyzed (20,000 cells/sample) in a FACSCanto flow cytometer (Benton & Dickinson) with the FITC filter (excitation 488 nm / emission 530 nm) and/or monitored by fluorescence microscopy in a Nikon Eclipse TE2000-E using the FITC filter (excitation 482 nm / emission 536 nm) with 1000 ms of exposition. Evaluation of the response of the *PHO89* promoter to external calcium was performed similarly but adding calcium to 0.6 M from a 4 M stock solution.

### Monitoring of ROS generation

*K. phaffii* X-33 cells exponentially growing in YPD medium were incubated for 1 h with dihydrorhodamine 123 (D1054, Sigma) at a final concentration of 2.5 µg/ml, as described in [7, 59]. Then, aliquots were taken and treated with KCl or KOH (20 and 30 mM, raising the pH to 7.4 and 8.1, respectively). After 30 min of incubation, samples were taken, cells fixed with formaldehyde as described above, and analyzed by fluorescence microscopy and flow cytometry.

### Electronic supplementary material

Below is the link to the electronic supplementary material.

**Additional file 1**. Supplemental Fig. 1 (ratio of expression of cells growing on glucose or glycerol prior shifting cells to alkaline pH), 2 (transcriptomic profiling of genes selected for construction of GFP reporters), 3 (Induction factor for selected genes based on RPKM values) and 4 (Growth of *K. phaffii* under moderate alkaline pH)

**Additional file 2**. Genes induced and repressed at least 2-fold

**Additional file 3**. Oligonucleotides used in this work

## Data Availability

The datasets generated and analyzed during the current study concerning RNA-seq are available at NCBI’s Gene Expression Omnibus (GEO) and can be retrieved under study GSE224427. Any other data are available from the corresponding author on reasonable request.
