# Correction: Transcriptomic profiling of the yeast Komagataella phaffii in response to environmental alkalinization

**DOI:** 10.1186/s12934-023-02210-2

**Published:** 2023-10-14

**Authors:** Marcel Albacar, Abdelghani Zekhnini, Jorge Pérez-Valle, José L. Martínez, Antonio Casamayor, Joaquín Ariño

**Affiliations:** 1 Institut de Biotecnologia i Biomedicina & Departament de Bioquímica i Biologia Molecular, Universitat Autònoma de Barcelona, Cerdanyola del Vallès, 08193 Spain; 2 Department of Biotechnology and Biomedicine, Section for Synthetic Biology, Technical University of Denmark, Kongens Lyngby, Denmark

**Microbial Cell Factories (2023) 22:63**

10.1186/s12934-023-02074-6

In the original publication of the article [1], the additional files 2 and 3 were swapped. The file under Additional file 2 should be Additional file 3 and vice-versa. The original article has been corrected.

### Electronic Supplementary Material

**Additional file 1.** Supplemental Fig. 1 (ratio of expression of cells growing on glucose or glycerol prior shifting cells to alkaline pH), 2 (transcriptomic profiling of genes selected for construction of GFP reporters), 3 (Induction factor for selected genes based on RPKM values) and 4 (Growth of K. phaffii under moderate alkaline pH)

**Additional file 2.** Genes induced and repressed at least 2-fold

**Additional file 3.** Oligonucleotides used in this work
