# Supplementary material for: Transcriptomic profiling of the yeast Komagataella phaffii in response to environmental alkalinization
Source: Microb Cell Fact. 2023 Apr 4;22:63. doi: 10.1186/s12934-023-02074-6 (PMC10071690; doi:10.1186/s12934-023-02074-6)
Supplement: Supplementary file 1 — Additional file 1. Supplemental Fig. 1 (ratio of expression of cells growing on glucose or glycerol prior shifting cells to alkaline pH), 2 (transcriptomic profiling of genes selected for construction of GFP reporters), 3 (Induction factor for selected genes based on RPKM values) and 4 (Growth of K. phaffii under moderate alkaline pH) [file 12934_2023_2074_MOESM1_ESM.pdf]

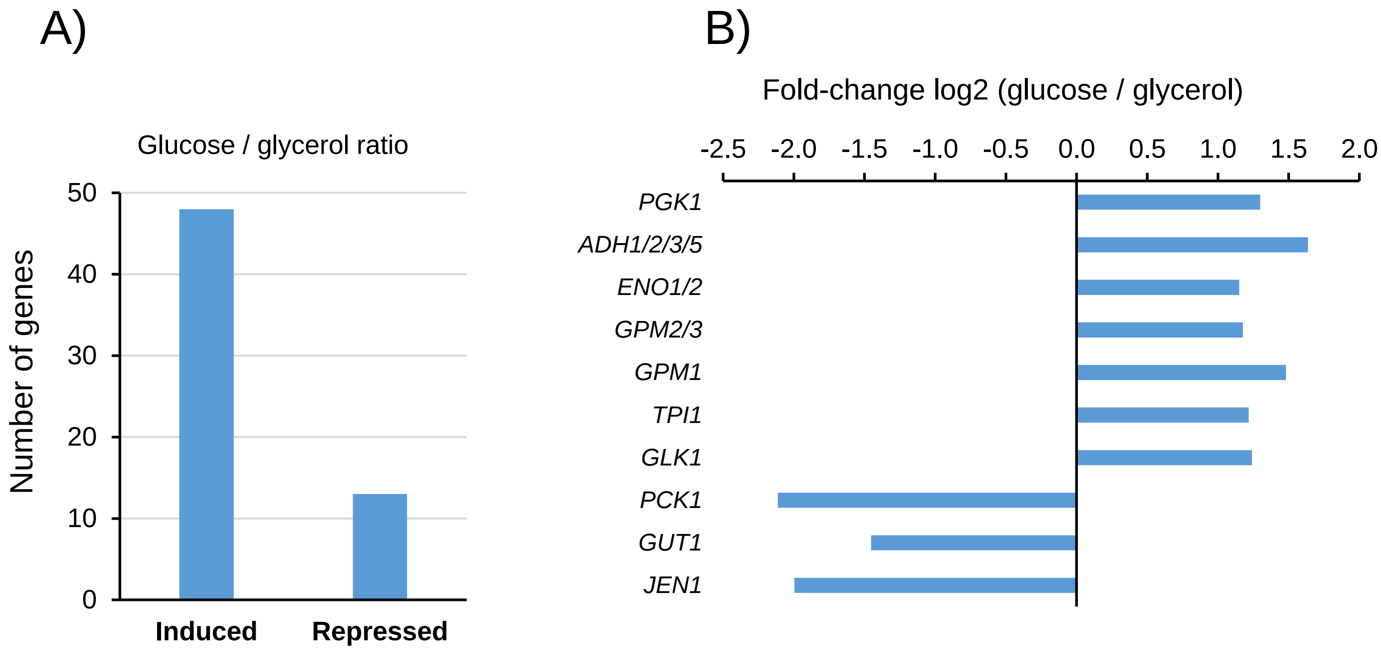

Supp. Figure 1. A) The ratio of expression of cells growing on glucose or glycerol was determined prior shifting cells to alkaline pH. Genes showing a log_2 ratio >= 1.0 or <= -1.0 were selected. B) Examples of induced genes, required for utilization of glucose, and of repressed genes, necessary for the use of glycerol (*GUT1*) or known to be repressed by glucose (*PCK1*, *JEN1*).

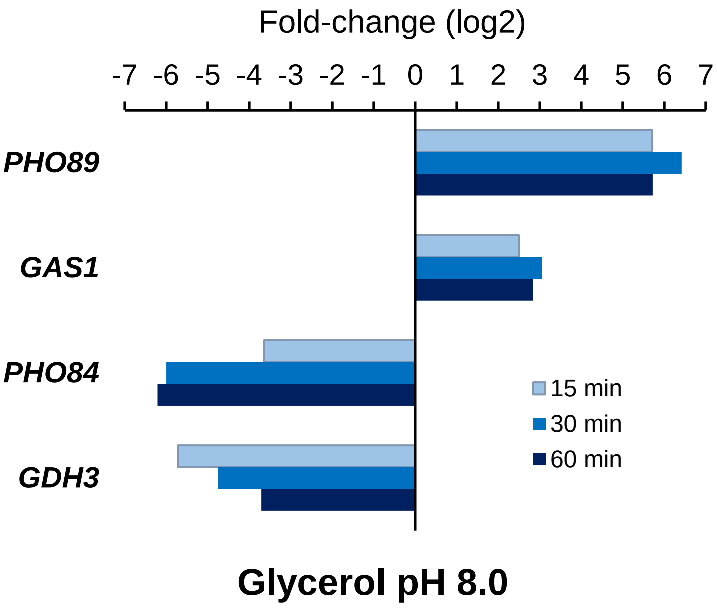

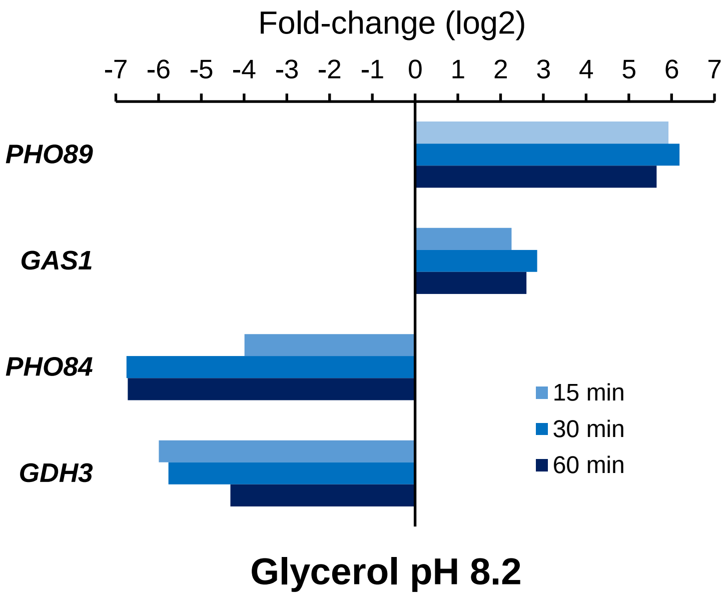

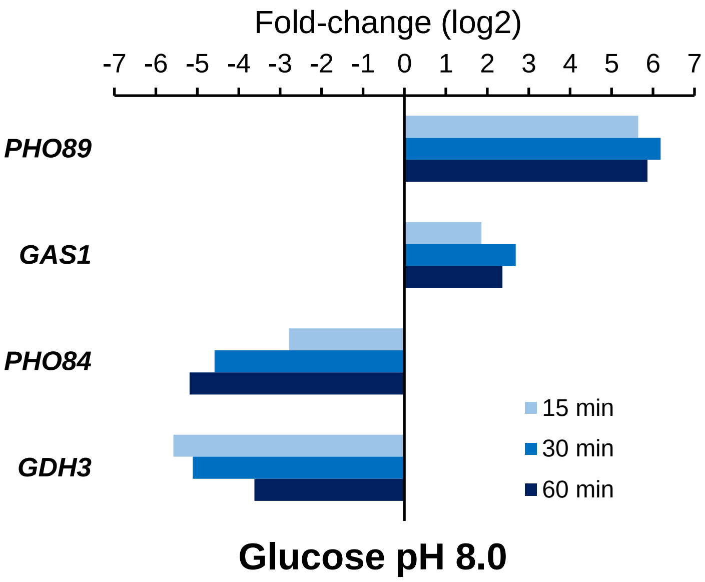

Supp. Figure 2. Transcriptomic profiling of genes selected for construction of GFP reporters. The specific experimental condition is indicated at the bottom of each graph.

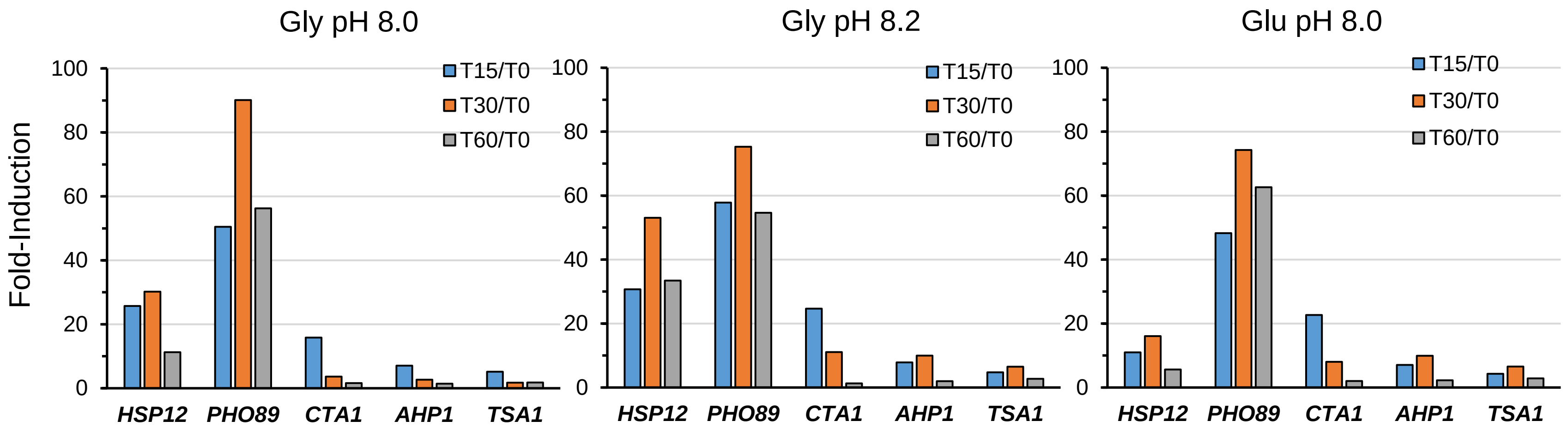

Supp. Figure 3. Induction factor for selected genes based on RPKM values. RPKM values for the indicated genes were determined for each time point and condition and the ratios versus time 0 were calculated and plotted.

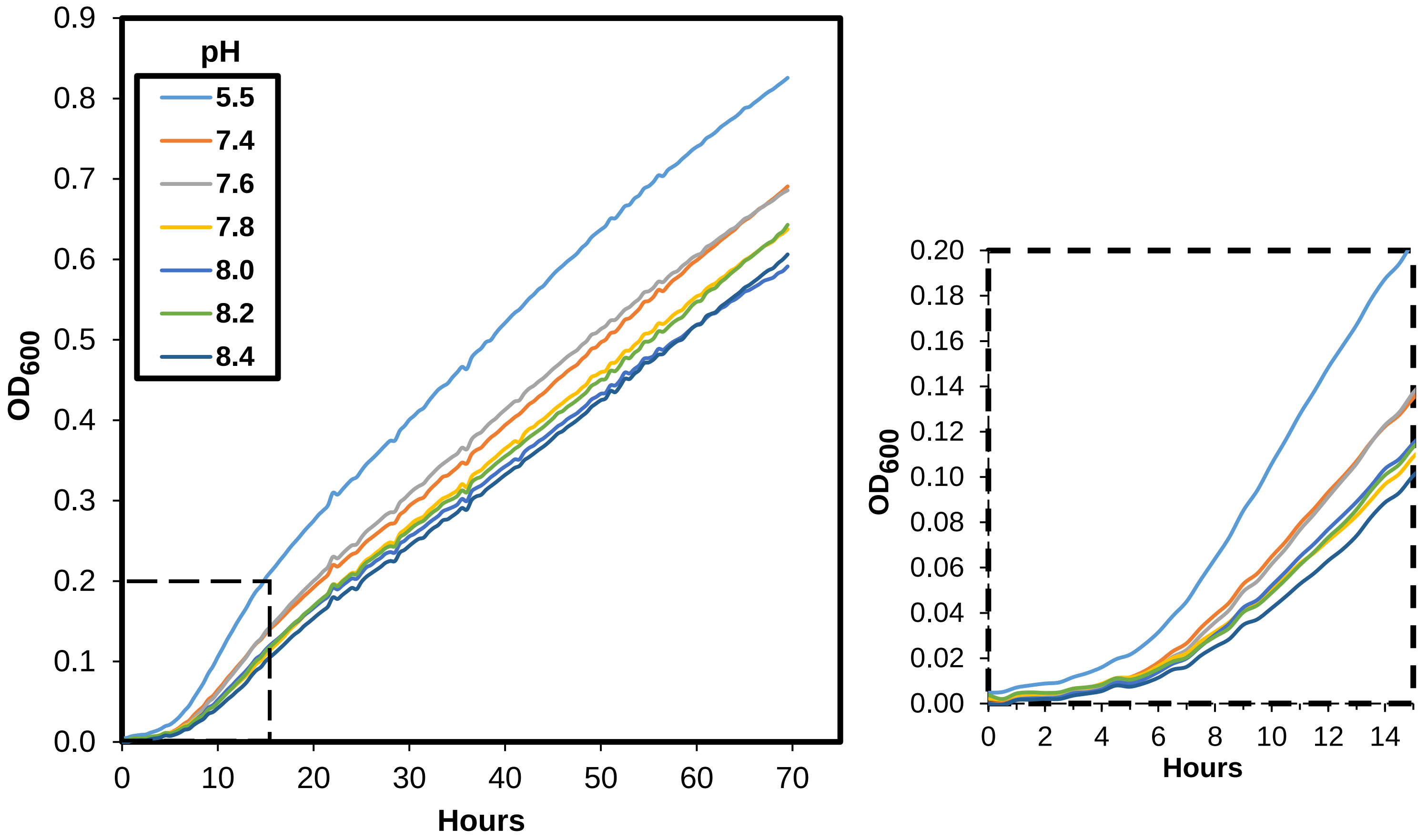

Supp. Figure 4. Growth of *K. phaffii* under moderate alkaline pH. One ml of fresh YPGly, containing 50 mM TAPS and adjusted to pH 5.5 was inoculated with saturated cultures of *K. phaffii* strain X-33 to OD_{600} =1,and then diluted to OD_{600} = 0.004 and transferred by triplicate to honeycomb plates (Thermo Fisher). Plates were introduced in a Bioscreen C apparatus (Thermo Fisher) and growth resumed at 28 °C with OD_{600} readings every 30 min. Data are mean from 6 biological replicates. SEM values are not included for clarity, and for most data points ranged from 2 to 4% of the corresponding density value. The small graph at the right is an expanded view of the first 15 h of culture.
